# Supplementary material for: A computed tomography vertebral segmentation dataset with anatomical variations and multi-vendor scanner data
Source: Sci Data. 2021 Oct 28;8:284. doi: 10.1038/s41597-021-01060-0 (PMC8553749; doi:10.1038/s41597-021-01060-0)
Supplement: Supplementary file 1 — Supplemental Table 1 [file 41597_2021_1060_MOESM1_ESM.pdf]

VerSe ID	Verse 2019	Verse 2020	gender	patient age (years)	CT scanner	Contrast enhancement	segmented cervical vertebrae (n)	segmented thoracical vertebrae (n)	segmented lumbar vertebrae (n)	vertebrae segmented (n)	data subset	cervical ribs (n)	thoracolumbar enumeration	thorakolumbar stump ribs	thoracolumbar transition vertebra	lumbosacral enumeration	Castellvi Grading	assumed segment structure
4	1	1	1	73,9	4	ce-no	0	4	5	9	1			0	0	0	0/1	7/12/5/5
5	1	1	1	74,2	1	ce-no	0	0	5	5	1			L1	1	0	0/1	7/12/5/5
6	1	0	0	78,9	2	ce-art	0	0	2	2	1					0	0/1	7/12/5/5
7	1	0	1	78	3	ce-no	0	4	3	7	1			0	0			7/12/5/5
8	1	1	1	59,8	1	ce-no	0	1	5	6	1			T12	2	0	0/1	7/12/5/5
9	1	0	1	88,2	1	ce-no	0	11	5	16	1	0	0	0	0	0	0/1	7/12/5/5
10	1	0	1	78,4	1	ce-no	0	0	5	5	2					0	0/1	7/12/5/5
11	1	0	1	70,9	1	ce-no	0	12	0	12	2	0	0	0	0			7/12/5/5
12	1	0	0	77,4	3	ce-no	0	3	3	6	3			0	0			7/12/5/5
13	1	1	1	60,4	2	ce-pv	0	11	5	16	2		0	0	0	0	0/1	7/12/5/5
14	1	0	1	72,3	1	ce-no	0	0	5	5	1			0	0	0	0/1	7/12/5/5
15	1	1	1	77,1	1	ce-no	0	1	5	6	1			T12	2	0	2a	7/12/5/5
16	1	1	1	65,1	2	ce-pv	0	12	5	17	2	0	0	T12	2	0	0/1	7/12/5/5
18	1	1	0	65,6	3	ce-pv	0	12	4	16	2	0	0	0	0	0	0/1	7/12/5/5
20	1	1	1	85,8	1	ce-no	0	12	5	17	3	0	0	0	0	0	2b	7/12/5/5
22	1	1	1	69,7	1	ce-no	0	0	5	5	2			T12	2	0	0/1	7/12/5/5
23	1	1	1	74,4	1	ce-no	0	3	5	8	2			L1	1	2	2b	7/12/6/5
24	1	1	0	75,9	1	ce-no	0	5	5	10	2			0	0	2	3b	7/12/6/5
26	1	0	0	89,3	1	ce-no	0	6	5	11	2			L1	1	0	0/1	7/12/6/5
29	1	1	1	50,6	2	ce-pv	0	12	5	17	3	0	0	0	0	0	0/1	7/12/5/5
30	1	0	1	73,3	1	ce-no	0	0	5	5	2			0	0	0	0/1	7/12/5/5
31	1	1	1	52,8	1	ce-no	0	0	5	5	1			L1	1	0	0/1	7/12/5/5
32	1	0	0	83,3	4	ce-no	0	1	5	6	3			0	0	0	0/1	7/12/5/5
33	1	1	1	56,8	4	ce-no	1	12	5	18	1	0	0	0	0	0	0/1	7/12/5/5
34	1	1	0	58,4	4	ce-no	0	3	5	8	1			L1	1	2	3b	7/12/6/5
36	1	0	0	61,8	1	ce-no	0	0	4	4	1					0	0/1	7/12/5/5
38	1	0	0	54,1	2	ce-no	0	2	5	7	3			0	0	0	0/1	7/12/5/5
40	1	0	1	56,2	4	ce-pv	0	4	5	9	3			0	0	0	0/1	7/12/5/5
41	1	1	1	60,5	3	ce-pv	1	12	5	18	2	0	0	0	0	0	2a	7/12/5/5
43	1	0	1	82	1	ce-no	0	11	5	16	1		0	0	0	0		7/12/5/5
46	1	0	1	78,6	1	ce-no	0	0	5	5	1			0	0	0	0/1	7/12/5/5
47	1	1	1	80,4	4	ce-no	0	12	5	17	2	0	0	T12	2	0	0/1	7/12/5/5
48	1	0	1	75,2	1	ce-no	0	0	4	4	1					0	0/1	7/12/5/5
50	1	1	0	51,2	2	ce-pv	0	12	5	17	3	0	0	0	0	0	0/1	7/12/5/5
51	1	0	1	70	2	ce-pv	0	3	5	8	1			0	0	0	0/1	7/12/5/5
53	1	0	1	86,3	1	ce-no	0	0	5	5	3			0	0	0	0/1	7/12/5/5
54	1	1	1	85,9	1	ce-no	0	0	5	5	3			L1	1	0	0/1	7/12/5/5
55	1	1	0	64,4	3	ce-pv	0	6	5	11	3			0	0	0	0/1	7/12/5/5
56	1	1	1	78,3	4	ce-no	0	2	5	7	1			T12	2	0	0/1	7/12/5/5
58	1	0	1	79,3	3	ce-no	0	1	5	6	2			0	0	0	0/1	7/12/5/5
59	1	0	0	61,6	1	ce-no	0	6	1	7	3			0	0			7/12/5/5
60	1	0	1	80	4	ce-no	0	1	5	6	1			0	0	0		7/12/5/5
61	1	0	0	70,7	3	ce-no	0	3	5	8	1			0	0	0	0/1	7/12/5/5
63	1	0	0	56,2	1	ce-no	0	0	5	5	1			0	0	0	0/1	7/12/5/5
64	1	0	1	56,8	1	ce-no	0	11	5	16	1		0	0	0	0	0/1	7/12/5/5
65	1	0	1	77,8	1	ce-no	0	0	4	4	1					0		7/12/5/5
66	1	1	1	57,1	1	ce-no	0	0	5	5	3			T12	2	0	2a	7/12/5/5
67	1	1	1	60,6	4	ce-pv	0	5	5	10	2			T12	2	0	2a	7/12/5/5
68	1	0	1	74,9	2	ce-pv	0	4	5	9	1			0	0	0	0/1	7/12/5/5
70	1	1	1	73,4	1	ce-no	0	4	5	9	3			L1	1	0	2b	7/12/5/5
71	1	0	1	57,2	3	ce-no	0	0	5	5	2					0	0/1	7/12/5/5
72	1	0	1	83,9	1	ce-no	0	1	5	6	1			0	0	0	0/1	7/12/5/5
73	1	1	1	75,5	1	ce-no	0	0	5	5	2			L1	1	0	0/1	7/12/5/5
74	1	0	0	63,9	3	ce-no	0	12	5	17	1	0	0	0	0	0	0/1	7/12/5/5
75	1	1	1	56,6	2	ce-pv	0	4	3	7	1			0	0	0		7/12/5/5
76	1	0	1	71,6	1	ce-no	0	0	5	5	1			0	0	0	0/1	7/12/5/5
78	1	0	1	80,3	1	ce-no	0	1	5	6	2			0	0	0	0/1	7/12/5/5
80	1	1	1	76,9	1	ce-no	0	10	5	15	2		0	0	0	0	0/1	7/12/5/5
81	1	0	1	81,3	1	ce-no	0	2	2	4	3			0	0			7/12/5/5
82	1	1	0	52,9	2	ce-no	0	12	5	17	1	0	0	0	0	0	0/1	7/12/5/5
83	1	1	1	62,8	4	ce-no	0	1	5	6	3			0	0	2	0/1	7/12/6/5
85	1	1	0	79,2	1	ce-no	0	0	5	5	3					2	3a	7/12/6/5
88	1	1	0	60,8	2	ce-no	0	4	5	9	1			L1	1	2	4	7/12/6/5
89	1	1	0	77,9	4	ce-no	0	2	5	7	3			0	0	0	0/1	7/12/5/5
91	1	1	1	77,2	2	ce-pv	0	12	5	17	1	0	0	T12	2	0	0/1	7/12/5/5
92	1	1	0	71,4	3	ce-pv	0	12	5	17	3	0	0	0	0	0	0/1	7/12/5/5
93	1	1	1	75	3	ce-no	0	4	5	9	2			0	0	0	0/1	7/12/5/5
95	1	0	1	67,1	4	ce-no	0	12	5	17	2	0	0	0	0	0	0/1	7/12/5/5
96	1	1	1	59,9	2	ce-art	1	12	5	18	1	0	0	0	0	0	0/1	7/12/5/5
97	1	1	1	90,2	3	ce-no	3	12	5	20	1	0	0	0	0	0	0/1	7/12/5/5
100	1	0	0	66,4	2	ce-no	0	4	4	8	1			0	0	0	0/1	7/12/5/5
101	1	1	1	52,7	2	ce-art	0	12	5	17	3	0	0	0	0	0	0/1	7/12/5/5
102	1	0	0	81,2	2	ce-no	0	0	5	5	1			0	0	0	0/1	7/12/5/5
104	1	1	1	66,7	1	ce-no	0	11	5	16	1		0	0	0	0	0/1	7/12/5/5
105	1	0	1	74,1	1	ce-no	0	9	0	9	1							7/12/5/5
107	1	0	1	74,1	3	ce-no	0	2	5	7	1			0	0	0	0/1	7/12/5/5
108	1	1	1	51,9	3	ce-no	7	12	5	24	3	0	0	T12	2	0	0/1	7/12/5/5
111	1	1	1	79,7	1	ce-no	0	2	5	7	1			T12	2	0	0/1	7/12/5/5
112	1	1	1	55,8	3	ce-pv	0	12	5	17	1	0	0	0	0	0	0/1	7/12/5/5
113	1	1	1	61,1	1	ce-no	0	0	5	5	1					2	0/1	7/12/6/5
116	1	0	0	66,3	3	ce-no	0	0	1	1	2					0	0/1	7/12/5/5
119	1	1	1	81	4	ce-no	0	12	1	13	3	0	0	0	0			7/12/5/5
122	1	1	1	80,1	1	ce-no	1	12	5	18	1	0	0	0	0	0	0/1	7/12/5/5
124	1	0	0	73,4	1	ce-no	0	0	5	5	2			0	0	0	0/1	7/12/5/5
125	1	1	1	84,8	2	ce-no	0	11	1	12	2	0	0	0	0			7/12/5/5
127	1	1	1	70,9	3	ce-pv	0	12	5	17	1	0	0	0	0	0	0/1	7/12/5/5
130	1	1	1	55,8	1	ce-no	0	5	5	10	3			0	0	0	0/1	7/12/5/5
131	1	1	1	70,6	4	ce-no	0	3	6	9	3			0	0	2	2a	7/12/6/5
133	1	0	1	67	3	ce-pv	0	4	5	9	1			0	0	0	0/1	7/12/5/5
134	1	0	1	67,1	1	ce-no	0	1	5	6	1			0	0	0	0/1	7/12/5/5
135	1	1	1	70,2	2	ce-pv	1	12	5	18	1	0	0	0	0	0	0/1	7/12/5/5
137	1	0	1	71,7	1	ce-no	0	1	5	6	1			0	0	0		7/12/5/5
138	1	1	0	58,8	2	ce-no	0	4	5	9	3			T12	2	0	2b	7/12/5/5
139	1	1	1	78,7	4	ce-no	0	0	5	5	1			T12	2	0	0/1	7/12/5/5
141	1	1	1	62,6	4	ce-no	1	12	5	18	1	0	0	L1	1	0	0/1	7/12/5/5
143	1	1	1	84,5	1	ce-no	0	0	5	5	3			T12	2	0	2a	7/12/5/5
144	1	0	0	75,4	1	ce-no	0	4	5	9	3			0	0	0	0/1	7/12/5/5
145	1	1	1	60,7	2	ce-pv	0	12	5	17	1	0	0	T12	2	0	0/1	7/12/5/5
146	1	0	1	70,2	1	ce-no	0	0	3	3	1					0	0/1	7/12/5/5
147	1	0	0	61,4	1	ce-no	0	0	5	5	3			0	0	0		7/12/5/5
149	1	1	1	88,9	1	ce-no	0	3	5	8	3			L1	1	0	0/1	7/12/5/5
150	1	0	0	76,3	1	ce-no	0	4	2	6	2			0	0			7/12/5/5
151	1	1	0	59,5	2	ce-art	0	12	5	17	1	0	0	0	0	0	0/1	7/12/5/5
152	1	0	1	74,2	1	ce-no	0	1	5	6	1			0	0	0	0/1	7/12/5/5
153	1	0	1	75,4	4	ce-no	0	4	5	9	2			0	0	0	0/1	7/12/5/5
154	1	1	0	56,3	3	ce-no	0	4	5	9	3			0	0	0	0/1	7/12/5/5
205	1	0	1	52,9	4	ce-no	7	0	0	7	2	0						7/12/5/5
207	1	0	1	90,6	1	ce-no	7	1	0	8	1	0						7/12/5/5

217	1	1	1	73,4	3	ce-no	7	2	0	9	3	0						7/12/5/5
221	1	0	0	76,5	3	ce-no	7	2	0	9	2	0						7/12/5/5
225	1	0	1	59,9	3	ce-no	7	2	0	9	2	0						7/12/5/5
230	1	0	1	79	3	ce-no	7	2	0	9	2	0						7/12/5/5
236	1	1	0	88,2	3	ce-no	7	12	5	24	3	0	0	0	0	0	0/1	7/12/5/5
242	1	0	0	68,6	5	ce-no	7	1	0	8	2	0						7/12/5/5
250	1	0	0	38,8	4	ce-no	7	2	0	9	3	0						7/12/5/5
252	1	1	0	36,1	4	ce-no	0	12	5	17	2	0	0	T12	2	0	3b	7/12/5/5
254	1	1	0	27,4	4	ce-no	1	12	5	18	1	0	0	L1	1	0	0/1	7/12/5/5
257	1	1	0	46,6	4	ce-no	0	3	5	8	1			0	0	0	0/1	7/12/5/5
260	1	1	1	58,1	4	ce-no	0	11	5	16	3		0	L1	1	0	0/1	7/12/5/5
264	1	1	1	29,3	3	ce-no	0	12	5	17	2	0	0	0	0	0	0/1	7/12/5/5
269	1	1	1	41,3	2	ce-no	0	11	5	16	2		0	0	0	0	0/1	7/12/5/5
271	1	1	0	49,7	3	ce-no	0	12	5	17	3	0	0	0	0	0	0/1	7/12/5/5
276	1	1	1	74,3	5	ce-no	1	12	5	18	2	0	0	L1	1	0	0/1	7/12/5/5
400	1	1	0	60,9	1	ce-no	7	11	5	23	2	0	0	0	0	0	0/1	7/12/5/5
401	1	1	1	58,6	1	ce-no	7	12	5	24	1	0	0	0	0	0	0/1	7/12/5/5
402	1	1	0	35,2	4	ce-no	7	12	5	24	1	0	0	0	0	0	0/1	7/12/5/5
403	1	1	0	53,6	5	ce-no	7	12	6	25	1	0	0	L1	1	2	0/1	7/12/6/5
404	1	1	0	59,1	4	ce-no	7	12	5	24	2	0	0	0	0	0	0/1	7/12/5/5
405	1	1	0	41,7	4	ce-no	7	12	5	24	1	0	0	0	0	0	0/1	7/12/5/5
406	1	1	0	40,7	4	ce-no	7	12	6	25	1	0		0	0	2	0/1	7/11,5/6/5
407	1	1	0	18,9	4	ce-no	7	12	5	24	1	0	0	0	0	0	0/1	7/12/5/5
408	1	1	1	74	2	ce-no	7	12	5	24	1	0	0	0	0	0	0/1	7/12/5/5
409	1	1	1	87	3	ce-no	7	12	5	24	1	0	0	0	0	0	0/1	7/12/5/5
410	1	1	1	64	2	ce-no	7	12	5	24	1	0	0	T12	2	0	0/1	7/12/5/5
411	1	1	1	61,4	3	ce-no	7	12	5	24	1	0	0	0	0	0	0/1	7/12/5/5
412	1	1	0	46,5	2	ce-no	7	12	5	24	2	0	0	0	0	0	0/1	7/12/5/5
413	1	1	1	23	2	ce-no	7	12	5	24	1	0	0	0	0	0	0/1	7/12/5/5
414	1	1	1	38,4	5	ce-no	7	12	5	24	3	0	0	0	0	0	0/1	7/12/5/5
415	1	1	0	34,8	4	ce-no	7	12	5	24	1	0	0	L1	1	0	0/1	7/12/5/5
416	1	1	0	52,6	4	ce-no	7	12	5	24	3	0	0	0	0	0	0/1	7/12/5/5
417	1	1	0	26,5	4	ce-no	3	12	5	20	3	0	0	0	0	0	2b	7/12/5/5
500	0	1	0	35	3	ce-pv	0	3	5	8	1			0	0	0	2a	7/12/5/5
502	0	1	1	35	4	ce-pv	0	12	5	17	3	0	0	0	0	2	3b	7/12/6/5
503	0	1	0	30	2	ce-no	0	2	4	6	1			0	0	0	3b	7/12/5/5
504	0	1	0	54	3	ce-no	0	2	5	7	1			0	0	0	2a	7/12/5/5
505	0	1	0	59	3	ce-pv	0	4	6	10	2	0		L1	1	2	0/1	7/12/6/5
506	0	1	0	55	3	ce-pv	1	12	6	19	1	0	0	T12	2	2	2b	7/12/6/5
507	0	1	1	69	3	ce-no	0	4	5	9	1	0		T12	2	0	2a	7/12/5/5
508	0	1	0	39	4	ce-art	0	12	5	17	2	0	0	0	0	0	2b	7/12/5/5
509	0	1	0	74	3	ce-pv	1	12	6	19	3	0	0	L1	1	2	2b	7/12/6/5
510	0	1	1	53	2	ce-pv	0	12	5	17	1	0	0	0	0	2	3a	7/12/6/5
511	0	1	0	58	3	ce-pv	0	11	5	16	2	0	0	0	0	0	0/1	7/12/5/6
512	0	1	1	68	3	ce-pv	1	12	5	18	3	0	0	0	0	2	3b	7/12/6/5
513	0	1	0	81	3	ce-pv	0	12	5	17	2	0	0	L1	1	2	3b	7/12/6/5
514	0	1	1	57	3	ce-pv	0	12	5	17	1	0	0	0	0	2	3b	7/12/6/5
517	0	1	1	42	3	ce-pv	0	11	6	17	3	0	0	0	0	2	0/1	7/12/6/5
518	0	1	0	74	3	ce-pv	0	12	5	17	1	0	0	0	0	2	4	7/12/6/5
519	0	1	0	71	3	ce-pv	0	11	6	17	1	0	0	0	0	2	2a	7/12/6/5
521	0	1	1	35	7	ce-no	1	12	5	18	1	0	0	0	0	0	2a	7/12/5/5
522	0	1	1	71	3	ce-pv	0	4	6	10	2	0		0	0	2	0/1	7/12/6/5
524	0	1	0	55	4	ce-pv	2	12	4	18	3	0	0	T12	2	0	3b	7/12/5/5
525	0	1	0	81	4	ce-no	0	0	6	6	1			L1	1	2	2a	7/12/6/5
526	0	1	0	80	3	ce-pv	0	12	5	17	3	0	0	0	0	2	3b	7/12/6/5
527	0	1	0	69	3	ce-pv	0	5	5	10	1			L1	1	2	3b	7/12/6/5
529	0	1	1	20	3	ce-pv	0	8	5	13	2			L1	1	2	3a	7/12/6/5
530	0	1	0	77	2	ce-no	0	2	6	8	2			0	0	2	2b	7/12/6/5
531	0	1	0	25	4	ce-pv	4	12	5	21	2	0	0	T12	2	0	0/1	7/12/5/5
532	0	1	0	23	4	ce-pv	0	3	6	9	1			0	0	2	0/1	7/12/6/5
533	0	1	0	41	4	ce-pv	0	11	5	16	1		0	0	0	0	0/1	7/12/5/6
534	0	1	0	75	3	ce-pv	0	3	5	8	1			0	0	2	3b	7/12/6/5
535	0	1	0	78	2	ce-pv	0	4	5	9	1			0	0	2	3b	7/12/6/5
536	0	1	0	56	3	ce-pv	0	6	5	11	1			L1	1	2	3b	7/12/6/5
537	0	1	1	54	3	ce-pv	0	3	5	8	1			0	0	0	2a	7/12/5/5
539	0	1	0	83	3	ce-pv	0	7	6	13	1			0	0	2	2b	7/12/6/5
540	0	1	1	19	3	ce-no	0	12	6	18	3	0	0	0	0	2	2a	7/12/6/5
541	0	1	1	28	3	ce-pv	0	3	5	8	1			0	0	0	0/1	7/12/5/5
542	0	1	1	77	2	ce-no	0	12	6	18	1	0	0	L1	1	2	2b	7/12/6/5
544	0	1	1	58	7	ce-art	0	10	2	12	1	0	1	0	0	2	3b	7/11/6/5
545	0	1	0	42	4	ce-no	0	2	5	7	2			0	0	2	4	7/12/6/5
546	0	1	0	45	3	ce-no	0	2	5	7	3			0	0	0	2b	7/12/5/5
547	0	1	1	24	3	ce-pv	7	13	5	25	2	0	2	T13	2	0	0/1	7/13/5/5
549	0	1	1	48	4	ce-pv	7	12	4	23	2	0	0	0	0	1	0/1	7/12/4/5
550	0	1	0	38	2	ce-pv	0	2	5	7	3			0	0	0	0/1	7/12/5/6
551	0	1	0	85	8	ce-art	0	11	6	17	3		0	L1	1	2	2b	7/12/6/5
552	0	1	0	67	3	ce-pv	0	12	4	16	3	0	0	0	0	1	0/1	7/12/4/5
553	0	1	1	60	3	ce-pv	0	11	6	17	2		0	0	0	2	2b	7/12/6/5
554	0	1	0	70	3	ce-art	0	11	5	16	2		0	L1	1	2	3b	7/12/6/5
555	0	1	0	43	2	ce-no	0	4	5	9	3			L1	1	2	4	7/12/6/5
556	0	1	0	33	3	ce-pv	0	5	5	10	2			0	0	2	3a	7/12/6/5
557	0	1	1	53	3	ce-pv	0	12	5	17	1	0	0	L1	1	2	3a	7/12/6/5
558	0	1	0	62	4	ce-no	2	12	5	19	3	0	0	0	0	0	2a	7/12/5/5
559	0	1	1	76	6	ce-pv	5	11	5	21	2	0	1	L1	1	1	0/1	7/11/4/5
560	0	1	0	68	6	ce-pv	0	12	6	18	3	0	0	0	0	2	0/1	7/12/6/5
561	0	1	1	40	6	ce-pv	5	12	5	22	1	0	0	T12	2	0	2a	7/12/5/5
563	0	1	1	68	4	ce-art	7	12	4	23	3	0	0	0	0	0	4	7/12/5/5
564	0	1	1	66	3	ce-art	0	4	5	9	1			T12	2	0	2b	7/12/5/5
565	0	1	0	48	2	ce-pv	0	4	6	10	1			0	0	2	2a	7/12/6/5
569	0	1	1	46	3	ce-no	1	12	6	19	2	0	0	0	0	2	0/1	7/12/6/5
570	0	1	0	50	3	ce-pv	0	12	6	18	3	0	0	0	0	2	2a	7/12/6/5
571	0	1	1	70	3	ce-pv	0	12	6	18	2	0	0	0	0	2	2a	7/12/6/5
572	0	1	1	22	4	ce-no	0	1	5	6	3			0	0	2	3a	7/12/6/5
573	0	1	1	37	2	ce-no	7	12	4	23	2	0	0	0	0	0	3a	7/12/5/5
576	0	1	1	36	3	ce-pv	0	12	6	18	2	0	0	L1	1	2	2a	7/12/6/5
577	0	1	0	57	4	ce-pv	1	12	6	19	1	0	0	0	0	2	2a	7/12/6/5
578	0	1	1	59	7	ce-no	0	4	6	10	3			0	0	2	2a	7/12/6/5
580	0	1	0	27	3	ce-no	0	3	6	9	2			0	0	2	2b	7/12/6/5
581	0	1	1	25	3	ce-no	7	11	5	23	1	1	1	L1	1	2	3b	7/11/6/5
582	0	1	0	51	4	ce-pv	3	13	5	21	3	0	2	T13	2	0	2a	7/13/5/5
584	0	1	1	21	3	ce-pv	0	4	4	8	1			T12	2	0	3b	7/12/5/5
585	0	1	0	35	6	ce-pv	7	12	6	25	2	0	0	0	0	2	0/1	7/12/6/5
586	0	1	0	70	3	ce-pv	0	12	6	18	1	0	0	0	0	2	2a	7/12/6/5
587	0	1	1	68	3	ce-no	1	12	5	18	3	0	0	L1	1	2	4	7/12/6/5
588	0	1	0	82	6	ce-pv	5	13	5	23	1	0	2	0	0	0	0/1	7/13/5/5
590	0	1	0	39	3	ce-pv	0	3	5	8	3			L1	1	2	3a	7/12/6/5
591	0	1	0	67	3	ce-pv	0	3	4	7	3			T12	2	0	3b	7/12/5/5
592	0	1	0	50	3	ce-no	0	3	5	8	2			0	0	2	3a	7/12/6/5
593	0	1	0	40	4	ce-no	3	12	6	21	1	0	0	L1	1	2	2a	7/12/6/5
594	0	1	0	30	3	ce-art	0	5	4	9	1			0	0	0	3b	7/12/5/5
596	0	1	0	62	6	ce-pv	0	10	0	10	1	1	0	T12	2	0	3b	7/12/5/5
597	0	1	0	64	2	ce-pv	0	2	6	8	3			0	0	2	2a	7/12/6/5
598	0	1	1	57	4	ce-pv	0	12	6	18	2	0	0	0	0	2	2b	7/12/6/5
599	0	1	0	58	4	ce-pv	3	12	5	20	3	0	0	L1	1	2	3a	7/12/6/5
600	0	1	0	51	3	ce-pv	0	3	5	8	2			0	0	0	2a	7/12/5/5

601	0	1	0	56	3	ce-pv	0	13	4	17	2	0	2	T13	2	0	3a	7/13/5/5
602	0	1	0	65	3	ce-no	7	13	5	25	3	0	2	T13	2	0	0/1	7/13/5/5
603	0	1	0	60	3	ce-pv	0	12	4	16	2	0	0	0	0	0	3a	7/12/5/5
604	0	1	1	58	3	ce-pv	0	3	6	9	3			0	0	2	0/1	7/12/6/5
605	0	1	0	38	4	ce-no	0	12	6	18	1	0	0	0	0	2	2b	7/12/6/5
606	0	1	1	58	3	ce-no	2	11	5	18	2	1	1	L1	1	2	3b	7/11/6/5
607	0	1	0	64	6	ce-pv	5	12	6	23	3	0	0	0	0	2	2a	7/12/6/5
609	0	1	0	31	3	ce-pv	0	1	5	6	2			L1	1	2	3a	7/12/6/5
612	0	1	0	47	3	ce-no	0	1	6	7	2			0	0	2	2a	7/12/6/5
613	0	1	0	43	4	ce-no	0	12	6	18	3	0	0	L1	1	2	0/1	7/12/6/5
614	0	1	1	35	2	ce-pv	0	4	6	10	2			L1	1	2	0/1	7/12/6/5
615	0	1	0	65	3	ce-art	0	11	6	17	2	0	0	T12	2	0	2b	7/12/5/5
616	0	1	1	77	3	ce-art	0	4	5	9	3			L1	1	2	3a	7/12/6/5
617	0	1	0	56	3	ce-art	0	3	4	7	3			T12	2	0	3b	7/12/5/5
618	0	1	0	28	2	ce-pv	3	12	5	20	2	0	0	0	0	2	4	7/12/6/5
619	0	1	1	80	3	ce-pv	0	12	6	18	1	0	0	0	0	2	2b	7/12/6/5
620	0	1	0	19	4	ce-no	1	12	6	19	3	0	0	L1	1	2	2b	7/12/6/5
621	0	1	0	57	3	ce-art	0	3	5	8	2			T12	2	0	2a	7/12/5/5
623	0	1	0	63	4	ce-pv	3	12	6	21	2	0	0	L1	1	2	2b	7/12/6/5
626	0	1	1	58	3	ce-pv	1	11	5	17	3	0	1	0	0	2	3b	7/11/6/5
627	0	1	1	47	3	ce-pv	0	4	4	8	2			T12	2	0	3b	7/12/5/5
629	0	1	1	22	4	ce-no	0	2	5	7	1			T12	2	0	2a	7/12/5/5
631	0	1	0	64	6	ce-pv	7	13	5	25	1	0	2	T13	2	0	2a	7/13/5/5
635	0	1	0	51	3	ce-no	0	3	5	8	2			T12	2	0	2a	7/12/5/5
636	0	1	1	42	3	ce-pv	1	11	5	17	2	0	1	0	0	2	3b	7/11/6/5
640	0	1	0	57	3	ce-no	1	12	1	14	2	1	0	T12	2		0/1	7/12/5/5
641	0	1	1	82	4	ce-no	7	2	0	9	1	1				0		7/12/5/5
642	0	1	1	32	4	ce-pv	3	11	6	20	1	1	1	0	0	2	2a	7/11/6/5
643	0	1	1	49	3	ce-no	2	8	0	10	2	1				0		7/12/5/5
644	0	1	1	29	4	ce-pv	7	12	5	24	2	1	0	0	0	0	0/1	7/12/5/5
645	0	1	1	50	3	ce-art	1	11	0	12	2	2	0			0		7/12/5/5
646	0	1	1	40	2	ce-pv	1	12	5	18	1	2	0	T12	2	0	0/1	7/12/5/5
647	0	1	1	40	4	ce-no	7	2	0	9	3	1			0	0		7/12/5/5
648	0	1	1	0	2	ce-no	7	5	0	12	3	1			0	0		7/12/5/5
649	0	1	0	48	6	ce-pv	7	12	5	24	3	1	0	0	0	0	0/1	7/12/5/5
650	0	1	1	86	1	ce-art	7	3	0	10	3	2			0	0		7/12/5/5
651	0	1	1	37	4	ce-no	7	2	0	9	3	2			0	0		7/12/5/5
700	0	1	0	35	10	ce-pv	0	6	5	11	3			0	0	0	0/1	7/12/5/5
701	0	1	1	64	10	ce-pv	0	3	5	8	2			0	0	0	0/1	7/12/5/5
702	0	1	0	77	10	ce-pv	0	4	5	9	3			0	0	0	0/1	7/12/5/5
703	0	1	0	32	10	ce-no	0	3	5	8	2			0	0	0	0/1	7/12/5/5
704	0	1	0	45	10	ce-art	0	12	5	17	3	0	0	0	0	0	0/1	7/12/5/5
705	0	1	1	62	10	ce-pv	0	12	5	17	2	0	0	0	0	0	0/1	7/12/5/5
706	0	1	1	37	10	ce-pv	0	12	5	17	3	0	0	0	0	0	0/1	7/12/5/5
707	0	1	0	53	10	ce-no	0	2	5	7	2			0	0	0	0/1	7/12/5/5
708	0	1	1	50	10	ce-pv	0	11	5	16	3	0	0	L1	1	0	0/1	7/12/5/5
709	0	1	1	85	10	ce-pv	0	3	5	8	2			0	0	0	0/1	7/12/5/5
710	0	1	0	73	10	ce-art	0	11	0	11	3		0	0	0	0		7/12/5/5
711	0	1	1	62	10	ce-pv	0	4	5	9	2			0	0	0	0/1	7/12/5/5
712	0	1	0	70	10	ce-art	0	4	5	9	3			0	0	0	0/1	7/12/5/5
713	0	1	1	67	10	ce-pv	0	5	5	10	2			0	0	0	0/1	7/12/5/5
714	0	1	0	66	10	ce-pv	0	4	5	9	3			0	0	0	0/1	7/12/5/5
715	0	1	1	70	10	ce-no	0	5	5	10	2			0	0	0	0/1	7/12/5/5
716	0	1	0	75	10	ce-art	0	3	5	8	3			0	0	0	0/1	7/12/5/5
717	0	1	0	53	10	ce-art	0	9	5	14	2			0	0	0	0/1	7/12/5/5
718	0	1	1	69	10	ce-no	0	2	5	7	3			0	0	0	0/1	7/12/5/5
719	0	1	1	71	10	ce-pv	0	2	5	7	2			0	0	0	0/1	7/12/5/5
750	0	1	1	51	9	ce-pv	0	11	5	16	2	0	0	0	0	0	0/1	7/12/5/5
751	0	1	0	58	9	ce-pv	0	6	6	12	3			L1	1	2	0/1	7/12/6/5
752	0	1	0	52	9	ce-art	0	11	5	16	3		0	0	0	0	0/1	7/12/5/5
753	0	1	1	28	9	ce-pv	0	3	5	8	2	0		0	0	0	0/1	7/12/5/5
754	0	1	0	45	9	ce-no	0	4	3	7	3			0	0	0		7/12/5/5
755	0	1	0	73	9	ce-pv	0	11	5	16	2		0	0	0	0	0/1	7/12/5/5
756	0	1	1	47	9	ce-pv	0	11	5	16	3		0	T12	2	0	0/1	7/12/5/5
757	0	1	0	21	9	ce-no	1	12	0	13	2	0	0	0	0	0		7/12/5/5
758	0	1	1	37	9	ce-no	7	12	5	24	3	0	0	0	0	0	0/1	7/12/5/5
759	0	1	0	83	9	ce-pv	7	12	5	24	2	1	0	T12	2	0	0/1	7/12/5/5
760	0	1	0	72	9	ce-no	0	3	5	8	3			0	0	0	0/1	7/12/5/5
761	0	1	0	36	9	ce-pv	0	11	5	16	2		0	0	0	0	0/1	7/12/5/5
762	0	1	1	59	9	ce-no	0	4	5	9	3	0		0	0	0	0/1	7/12/5/5
763	0	1	0	19	9	ce-no	0	4	5	9	2			0	0	0	0/1	7/12/5/5
764	0	1	1	73	9	ce-art	0	10	0	10	3	0	0	0	0	0		7/12/5/5
765	0	1	0	56	9	ce-pv	0	11	6	17	2	0	1	L1	1	2	0/1	7/11/6/5
766	0	1	0	78	9	ce-pv	7	12	5	24	3	0	0	0	0	0	2b	7/12/5/5
767	0	1	1	70	9	ce-no	0	12	0	12	2	0	0	0	0	0		7/12/5/5
768	0	1	0	56	9	ce-pv	0	5	6	11	3			L1	1	2	2b	7/12/6/5
769	0	1	0	48	9	ce-pv	0	5	5	10	2			0	0	0	0/1	7/12/5/5
801	0	1	0	53	8	ce-no	0	2	5	7	3			0	0	0	0/1	7/12/5/5
802	0	1	0	76	8	ce-art	0	6	5	11	2			0	0	0	0/1	7/12/5/5
803	0	1	1	52	8	ce-art	0	12	4	16	3	0	0	0	0	0	3b	7/12/5/5
804	0	1	1	35	8	ce-no	0	5	5	10	3			0	0	0	0/1	7/12/5/5
805	0	1	1	44	8	ce-pv	0	4	5	9	2			0	0	0	0/1	7/12/5/5
806	0	1	0	69	8	ce-pv	0	3	5	8	2			0	0	0	0/1	7/12/5/5
807	0	1	0	59	8	ce-pv	0	3	5	8	1	0		L1	1	0	0/1	7/12/5/5
808	0	1	1	71	8	ce-pv	0	3	5	8	1			0	0	0	2a	7/12/5/5
809	0	1	0	77	8	ce-pv	7	12	5	24	3	0	0	0	0	0	0/1	7/12/5/5
810	0	1	0	46	8	ce-pv	0	3	6	9	3			L1	1	2	0/1	7/12/6/5
811	0	1	0	49	8	ce-pv	0	4	5	9	1			0	0	0	0/1	7/12/5/5
813	0	1	1	59	8	ce-pv	0	4	5	9	3			0	0	0	0/1	7/12/5/5
814	0	1	1	77	8	ce-art	1	11	5	17	2	2	1	0	0	2	3b	7/11/6/5
815	0	1	0	64	8	ce-pv	0	2	5	7	2			0	0	0	0/1	7/12/5/5
816	0	1	1	56	8	ce-pv	0	3	6	9	2			0	0	2	0/1	7/12/6/5
817	0	1	1	56	8	ce-no	0	4	5	9	2			0	0	0	0/1	7/12/5/5
818	0	1	0	66	8	ce-pv	0	4	5	9	1			0	0	0	0/1	7/12/5/5
820	0	1	0	30	8	ce-pv	0	3	5	8	1			0	0	0	0/1	7/12/5/5
821	0	1	0	71	8	ce-pv	0	11	5	16	2		0	0	0	0	0/1	7/12/5/5
823	0	1	0	75	8	ce-pv	7	12	5	24	1	0	0	0	0	0	0/1	7/12/5/5
824	0	1	1	51	8	ce-pv	0	4	5	9	1			T12	2	0	0/1	7/12/5/5
825	0	1	0	67	8	ce-art	0	3	5	8	1			0	0	0	0/1	7/12/5/5
826	0	1	1	58	8	ce-pv	0	11	5	16	2	0	0	0	0	0	0/1	7/12/5/5
833	0	1	0	83	8	ce-art	0	6	5	11	1			0	0	0	2b	7/12/5/5
835	0	1	0	59	8	ce-no	0	2	5	7	1	2		0	0	0	0/1	7/12/5/5
Gl. 003	0	1	0	57	11	ce-no	7	5	0	12	1	0						7/12/5/5
Gl. 016	0	1	0	43	11	ce-no	7	4	0	11	1	0						7/12/5/5
Gl. 017	0	1	0	62	11	ce-no	7	4	0	11	2	0						7/12/5/5
Gl. 045	0	1	0	49	11	ce-no	7	4	0	11	2	0						7/12/5/5
Gl. 047	0	1	1	45	11	ce-no	7	4	0	11	1	0						7/12/5/5
Gl. 059	0	1	1	22	11	ce-no	7	3	0	10	2	0						7/12/5/5
Gl. 068	0	1	0	53	11	ce-no	7	5	0	12	2	0						7/12/5/5
Gl. 090	0	1	1	50	11	ce-no	7	3	0	10	1	0						7/12/5/5
Gl. 099	0	1	1	16	11	ce-no	7	7	0	14	2	0						7/12/5/5
Gl. 108	0	1	0	64	11	ce-no	7	3	0	10	3	0						7/12/5/5
Gl. 124	0	1	0	23	11	ce-no	7	3	0	10	1	0						7/12/5/5
Gl. 144	0	1	0	57	11	ce-no	7	4	0	11	2	0						7/12/5/5
Gl. 146	0	1	1	52	11	ce-no	7	5	0	12	3	0						7/12/5/5

**Gl. 153**	**0**	**1**	0	61	11	ce-no	7	3	0	10	2	0						7/12/5/5
**Gl. 195**	**0**	**1**	0	74	11	ce-no	7	4	0	11	3	0						7/12/5/5
**Gl. 216**	**0**	**1**	0	62	11	ce-no	7	2	0	9	3	0						7/12/5/5
**Gl. 217**	**0**	**1**	0	28	11	ce-pv	7	3	0	10	3	0						7/12/5/5
**Gl. 240**	**0**	**1**	0	59	11	ce-no	7	3	0	10	1	0						7/12/5/5
**Gl. 247**	**0**	**1**	0	73	11	ce-no	7	6	0	13	1	0						7/12/5/5
**Gl. 279**	**0**	**1**	0	23	11	ce-pv	0	11	6	17	3		0	L1	1	2	0/1	7/12/6/5
**Gl. 295**	**0**	**1**	0	27	11	ce-no	7	5	0	12	1	0						7/12/5/5
**Gl. 348**	**0**	**1**	0	59	11	ce-no	7	2	0	9	3	0						7/12/5/5
**Gl. 352**	**0**	**1**	0	73	11	ce-no	7	5	0	12	2	0						7/12/5/5
**Gl. 364**	**0**	**1**	1	48	11	ce-no	7	1	0	8	1	0						7/12/5/5
**Gl. 380**	**0**	**1**	0	49	11	ce-pv	7	3	0	10	2	0						7/12/5/5
**Gl. 419**	**0**	**1**	1	83	11	ce-myelo	7	4	0	11	3	0						7/12/5/5
**Gl. 428**	**0**	**1**	1	71	11	ce-no	7	6	0	13	3	0						7/12/5/5
**Gl. 453**	**0**	**1**	0	41	11	ce-no	7	3	0	10	1	0						7/12/5/5
**Gl. 479**	**0**	**1**	0	45	11	ce-no	7	4	0	11	2	0						7/12/5/5
**Gl. 492**	**0**	**1**	1	48	11	ce-no	7	4	0	11	3	0						7/12/5/5

Supplemental Table 1 lists the main characteristics of all VerSe (2019 and 2020) datasets on a patient level.

gender (0=m; 1=w)
CT-Scanner (1: Philips Brilliance 64; 2: Philips ICT; 3: Philips IQON, 4: Siemens Somatom AS+, 5 Siemens Somatom AS, 6: Siemens Biograph 64, external;
7: Siemens Sensation Cardiac 64; 8 Siemens external; 9: GE external; 10 Toshiba; 11: unknown from Glocker dataset)
Data subset (1: Public Training Subset, 2: Public Validation Subset, 3: Private Test Subset)
Thoracolumbar transition vertebra (0: non-anomalous, 1: lumbar-type transitional vertebra, 2: thoracic-type transitional vertebra)
Lumbosacral enumeration (0: 5 lumbar vertebrae L5/S1, 1: 4 lumbar vertebrae L4/S1, 2: 6 lumbar vertebraeL6/S1)
Thoracolumbal enumeration (0: 12 thoracic vertebrae T12/L1, 1: 11 thoracic vertebrae T11/L1, , 2: 13 thoracic vertebrae T13/L1)

Note: Double-segmented vertebral bodies of a subject due to overlapping scans were not counted more than once in this supplementary table.
